# Feedback in Medical Education: An Evidence-based Guide to Best Practices from the Council of Residency Directors in Emergency Medicine

**DOI:** 10.5811/westjem.56544

**Published:** 2023-05-05

**Authors:** Sreeja Natesan, Jaime Jordan, Alexander Sheng, Guy Carmelli, Brian Barbas, Andrew King, Kataryza Gore, Molly Estes, Michael Gottlieb

**Affiliations:** * Duke University, Department of Emergency Medicine, Durham, North Carolina; † David Geffen School of Medicine at UCLA, Department of Emergency Medicine, Los Angeles, California; ‡ Boston Medical Center, Department of Emergency Medicine, Boston, Massachusetts; § University of Massachusetts, Department of Emergency Medicine, Worcester, Massachusetts; ¶ Rush University Medical Center, Department of Emergency Medicine, Chicago, Illinois; || Loyola University Chicago, Stritch School of Medicine, Loyola University Medical Center, Department of Emergency Medicine, Maywood, Illinois; # The Ohio State University Wexner Medical Center, Department of Emergency Medicine, Columbus, Ohio; ** Loma Linda University, Department of Emergency Medicine, Loma Linda, California

## Abstract

Within medical education, feedback is an invaluable tool to facilitate learning and growth throughout a physician’s training and beyond. Despite the importance of feedback, variations in practice indicate the need for evidence-based guidelines to inform best practices. Additionally, time constraints, variable acuity, and workflow in the emergency department (ED) pose unique challenges to providing effective feedback. This paper outlines expert guidelines for feedback in the ED setting from members of the Council of Residency Directors in Emergency Medicine Best Practices Subcommittee, based on the best evidence available through a critical review of the literature. We provide guidance on the use of feedback in medical education, with a focus on instructor strategies for giving feedback and learner strategies for receiving feedback, and we offer suggestions for fostering a culture of feedback.

## BACKGROUND

Feedback is an important tool within medical education for the improvement of clinical skills and professional development.^1^ However, the emergency department (ED) presents a uniquely complex environment for feedback due to the rapid pace and workflow for patient care, relative lack of privacy, and need for constant task-switching.^1^ Incorporating feedback into this environment can negatively impact an emergency medicine (EM) resident’s training, with consistent reports of dissatisfaction regarding the quality of feedback received from faculty.^2^ The Accreditation Council for Graduate Medical Education (ACGME) Milestones stipulate that important domains for high-quality feedback should include timeliness, specificity, balance, recipient feedback/reflection, and an action plan.^1^,^3^–^30^

Despite the importance of feedback, evidence to inform best practices in the ED is limited, and there is a need for evidence-based guidelines to optimize feedback within the ED setting.^2^,^31^,^32^ Based on the best available evidence through a critical review of the literature, we offer expert guidelines on feedback from members of the Council of Residency Directors in Emergency Medicine (CORD) Best Practices Subcommittee. This paper provides readers with recommendations on the use of feedback, with a focus on giving and receiving feedback, and suggestions for fostering a positive culture of feedback.

## CRITICAL APPRAISAL

This is the tenth article in a series of evidence-based best practice reviews from the CORD Best Practices Subcommittee.^33^–^41^ Created for medical educators, these best-practice reviews cover a wide breadth of topics from clinical teaching, didactics, and journal club to guidance for increasing diversity, equity, and inclusion (DEI) initiatives for faculty and resident recruitment. We conducted a literature search in conjunction with a medical librarian using MEDLINE with a combination of medical subject heading (MeSH) terms and keywords focused on feedback searching for articles published from inception to March 15, 2021 (Appendix). We also reviewed the bibliographies of all included articles. Two authors (SN, MG) independently screened and included articles that addressed delivering feedback, receiving feedback, or feedback culture. We included articles based on discussion and negotiated consensus. Articles were excluded if they were not related to the three domains of feedback. The search yielded 2,402 articles, of which 207 were deemed to be directly relevant to this review. The level and grade of evidence were provided for each best-practice statement implementing the Oxford Centre for Evidence-Based Medicine criteria (Tables 1 and 2).^42^ When supporting data was not available, we made recommendations based upon our combined experience and consensus opinion drawn from expertise in research and scholarship regarding feedback and medical education. Prior to submission, our manuscript was peer reviewed by the CORD Best Practices Subcommittee and posted to the CORD website for two weeks for review by the entire CORD medical education community. We reviewed the comments and feedback prior to incorporating them into the final manuscript.

## GIVING FEEDBACK

### Components and Characteristics of High-quality Feedback

Delivering feedback is a complex process with many influencing factors. Prior literature has demonstrated that feedback practices by faculty vary.^31^,^44^ Educational experts, learners, and regulatory bodies agree on several key components and characteristics of high-quality feedback. They recommend that feedback should be clear, specific, timely, and actionable.^1^,^3^–^22^,^24^–^30^,^45^

Clarity in feedback is essential; lack of learner understanding leads to an inability to incorporate feedback into an action plan for improved performance.^45^ For feedback to be effective and valued by learners, it should be specific and based on directly observed behaviors and encounters. 1,9,13,14,24,46–55 In today’s era of competency-based medical education, it is important that feedback be targeted toward learner goals and a shared mental model of competencies and expectations.^4^,^6^,^11^,^22^,^29^,^47^,^56^–^59^ Feedback should be given using descriptive, non-judgmental language.^14^,^15^,^22^,^48^,^60^ It is important to target feedback toward actions and behaviors rather than judgment of the individual.^17^,^29^,^61^,^62^ Doing so has the benefit of mitigating the shame response in learners, which can worsen performance and feedback efficacy.^10^,^57^,^60^

Experts and learners advocate for feedback to be timely, which increases the likelihood that the feedback will be used for improvement.^3^–^7^,^20^,^26^,^28^,^30^,^52^,^54^,^63^ While finding time to provide feedback during clinical work can be a challenge,^46^,^64^–^67^ real-time feedback has been shown to improve performance. 68,69 Additionally, there is literature to support that real-time, workplace-based assessments provide more specific and effective feedback than end-of-rotation evaluations.^70^ The optimal volume and frequency of feedback are unknown. Multiple observations are likely required to achieve reliable assessments.^71^ Regular feedback is important to improve performance,^72^,^73^ and learners appreciate receiving frequent feedback.^6^,^21^ Some experts recommend that more feedback is necessary for the current generation of learners.^61^ It is important to note, however, that many learners may value quality over quantity in feedback.^8^

Constructive feedback is important and can lead to motivational learning and enhanced future performance.^74^ While some learners may value constructive feedback over reinforcing or encouraging feedback, both have been shown to be valuable.^10^,^18^,^29^,^30^,^75^–^78^ It may not be necessary or helpful to include both constructive and reinforcing feedback during the same conversation.^79^ Giving constructive feedback may be particularly challenging due to fear of retaliation (especially in systems where learner feedback is tied to summative evaluations, linked to author, pay or promotion).^80^,^81^ However, limited literature suggests that the fear of retaliation may be unwarranted.^82^,^83^ Other potential concerns surrounding constructive feedback include damaging rapport with learners or triggering an emotional response from trainees; however, these can be reduced by ensuring the feedback is clear, focused on actions or behaviors (as opposed to the individual), and supported by specific examples.^2^,^84^

As a step toward improved performance, incorporating co-creation of goals^58^,^85^–^87^ and the formation of learning or action plans into feedback can increase the benefit to trainees.^1^,^6^,^12^,^17^,^21^–^23^,^60^,^78^,^87^,^88^ Additionally, encouraging collaborative discussion and learner reflection during the feedback conversation may be beneficial.^7^,^12^,^48^,^60^,^74^,^89^ Faculty should be attentive and dedicated to providing feedback, as faculty effort and engagement have been shown to improve feedback.^7^,^90^

The setting in which feedback is given is also important.^5^,^26^ Feedback should be given in a non-threatening and supportive environment.^15^,^17^,^25^,^29^,^48^,^74^,^91^ It may be prudent to use different types of settings for different feedback activities.^4^ For example, constructive feedback may best be given in a one-on-one setting after a clinical encounter, whereas positive feedback on physical exams, procedural skills, or clinical decision-making may be more effective if given during or immediately after the patient encounter.^4^,^92^ The optimal method used to provide feedback is unknown, and strengths and weaknesses of various forms of feedback have been highlighted.^19^,^90^ Verbal feedback may be more helpful for engaging in collaborative discussion, but written feedback is more easily recognized and can serve as a reference for future reflection.^19^,^90^ It is important to note that inconsistencies exist between verbal and written feedback. An example is when learners receive positive laudatory feedback verbally, only to find disparaging or negative critiques in the written review. This inconsistency can lead to distrust and frustration among the learner and should be avoided.^47^

### Sources of Feedback and Personnel Involved

The source of feedback and the individuals involved can also impact feedback quality.^88^,^93^ It is important that feedback be from a credible source. 94 Learners consider feedback more valuable and credible when given by those they consider experts in that specific domain.^8^,^21^,^94^,^95^ However, the reliability of assessment may vary with assessor groups for different skills assessed; so, it can be valuable to deliberately align assessment and feedback areas with rater domains of expertise when possible.^71^ The relationship between the individuals involved in the feedback discussion is also important. Having a good relationship based on mutual respect and trust can enhance the quality and accuracy of feedback.^3^,^12^,^57^,^62^,^77^,^87^,^90^,^96^–^100^

Training individuals on how to give feedback can also improve the quality and specificity of feedback delivered.^32^,^48^,^56^,^91^,^101^–^107^ Training can lead to improved comfort with providing feedback and increase the likelihood of the learner incorporating reflection and goal-setting into feedback discussions.^108^,^109^ This is important, as lack of training in those providing feedback has been highlighted as a barrier to giving meaningful feedback.^20^

Feedback may come from multiple sources, and prior literature has demonstrated that both learners and supervisors value multisource feedback (MSF) as described in Table 1.^49^,^52^,^63^,^110^–^119^ Limited literature supports that MSF may be more helpful for identifying strengths and weaknesses compared to standard assessment methods and may be more likely to result in behavior change.^52^,^63^ Multisource feedback may also be effective in distinguishing between high, intermediate, and low performance in learners.^113^ Additionally, data on the correlations of assessments between assessor groups is mixed, and different assessor groups may provide distinct feedback.^63^,^71^,^110^,^120^–^128^ Variations in assessments between assessor groups could suggest that assessments may be different but not necessarily less valuable, lending support to the importance of having multiple perspectives in feedback systems to provide learners with more comprehensive data about their skills.^63^,^120^,^122^ Moreover, learners may value feedback from various groups differently^51^; so, care should be taken to align assessor qualifications with the assessments they will be performing.

Barriers to MSF do exist and include lack of training in those providing feedback, time and resources required to gather MSF, and the ability of learners to incorporate this type of feedback.^20^,^112^,^124^,^129^ Multisource feedback can be gathered synchronously or asynchronously,^121^ but regardless of route, it should be timely and ideally incorporate multiple settings.^20^,^63^ The incorporation of learner self-assessment into feedback can also have a positive impact.^17^,^22^,^23^,^128^,^130^,^131^

### Techniques and Tools for Providing Feedback

Currently, there is no consensus regarding the best methods for feedback and no formal endorsement by educational bodies of a single strategy.^18^ When providing feedback, it is important to use a variety of techniques and tools tailored to the individual learner and situation. We summarize several feedback techniques including direct observation, real-time feedback, self-assessment, multiple sources, and other specialized techniques in Table 3.

Each of these techniques has strengths and weaknesses. Direct observation has been shown to be highly valued and can increase clinical knowledge, skills, and attitudes; however, there is limited data to suggest a behavioral change.^132^ Strengths of direct observation include the emphasis on timely, learner-centered feedback.^107^ Challenges to direct observation include resources required, competing time demands of faculty and learners, perceived loss of credibility with patients by learners, and the Hawthorne effect.^46^,^132^–^135^ These barriers may be overcome by creating a structured, longitudinal direct observation and feedback program. 136 Real-time feedback is highly learner-centered, has been shown to improve the quantity of feedback given, and is generally well liked by users.^137^ However, it doesn’t necessarily improve feedback quality; studies have shown that less than 20% of the feedback given in real time is specific or corrective, often only focusing on positive and encouraging aspects of care.^27^,^138^

While learner self-assessment may not correlate well with external assessments,^122^,^127^,^128^ it can contribute positively to feedback discussions by encouraging reflection and establishing a shared understanding and mental model for feedback.^17^,^22^,^23^,^74^,^85^,^128^,^130^,^131^,^139^ Combining self-assessment with feedback can positively impact improvement behaviors.^130^,^131^ Importantly, while evaluative models for feedback have been shown to improve the number of feedback evaluations, they may not improve the quality of corrective feedback. 140,141 Multisource feedback tools are generally well liked and have good efficacy for competencies such as inter-professional communication and professionalism; however, they may be limited in their ability to identify struggling learners.^142^ Overall these techniques are quick and efficient and can work for a wide variety of learners to provide formative feedback.^143^

### Tools for Giving Feedback

Much like the variety of techniques for giving feedback, many tools have been developed to assist in providing feedback. Feedback tools have been demonstrated to increase the number of feedback encounters and improve learner satisfaction with feedback.^7^,^32^,^59^,^155^–^157^ However, it is important to note that feedback tools are not a replacement for verbal feedback or preceptor experience.^7^,^19^ We provide a summary of physical and electronic feedback tools including feedback cards, mini-cards, field note tool, MSF tools, web-based platforms, apps, crowdsourcing, and video recording in Table 4.

Like the techniques described above, each tool has its own strengths and weaknesses. Feedback encounter cards have repeatedly been shown to increase the perceived number of feedback encounters and, typically, improve learner satisfaction of quality, amount, and timeliness of feedback.^2^,^32^,^155^,^156^,^158^ However, some studies have reported that feedback may not be specific enough.^156^,^159^ This challenge can be mitigated by pairing encounter cards with a curriculum for educators and learners regarding giving and receiving feedback.^2^,^32^ Mini-cards and the Mini-Clinical Examination Exercise can identify the struggling learner and provide formative assessments to support their growth.^88^,^148^,^160^–^162^ Both tools can be integrated into routine clinical work while providing reliable assessments if at least 6–8 such encounters are used.^162^ A limitation noted for these card-based observation tools is that they may be perceived as a one-way evaluation and less likely to result in a learner-driven action plan. 148,161 As MSF has become more incorporated into feedback approaches, several tools have been developed and studied as listed in Table 3.^112^,^121^,^122^,^124^,^129^,^142^

With the increased availability of smartphones and portable devices, an array of new electronic-based feedback tools have been created and implemented with the hope of making the administration of feedback more convenient, accessible, and timely for educators and learners.^18^ Studies have shown that these web-based tools can be beneficial for improving faculty engagement in and frequency of their feedback.^11^,^163^,^164^ The timely nature of this feedback also leads to increased satisfaction from learners. 164 However, these platforms can be limited by faculty comfort with, and knowledge of, technology.^165^ Additionally, specific and corrective feedback may be challenging.^27^,^138^

It is important to consider data consolidation and distribution with these tools to ensure that feedback is distributed in a timely manner.^137^,^166^ To improve the accessibility of online feedback tools, several platforms have used quick response (QR) codes.^163^,^167^ The use of QR codes to access online feedback forms was found to be user-friendly and resulted in faster completion than paper and online web-based tools not associated with a QR code.^167^ Various apps have been created, which have led to an increase in the quality of feedback.^18^,^70^,^168^–^170^ Additional strengths include accessibility, low cost, and ability to trend resident progression.^18^ However, much like web-based platforms, app-based platforms can be limited by faculty and resident engagement.^18^,^170^ When instituting any app-based evaluation tool, it is important to pair it with training on the app and changes to feedback culture, such as regular encouragement, incentivization, physician champions, or regular reminders.^18^,^168^,^170^

Using online social media platforms (eg, Twitter messaging) is another tool to increase the volume and timeliness of feedback; however, effectiveness may be limited.^18^,^171^ Video-assisted feedback can be a valuable tool for feedback similar to direct observation.^133^,^172^ However, much like other forms of direct observation, video recording may not represent true, real-world encounters as learners may act differently due to the Hawthorne effect. Additionally, video recording can cause anxiety in trainees.^133^

Inviting Co-workers to Evaluate Physicians Tool (INCEPT); Mini Peer Assessment Tool (Mini-PAT); Team Assessment of Behavior (TAB); Emergency Medicine Humanism Scale (EM-HS); Communication Assessment Tool (CAT); Observed Structured Teaching Exercises (OSTE), or an Objective Structured Clinical Examination (OSCE) are other useful evaluation tools.

**Best Practice Recommendations**

1. **Feedback should be clear, specific, timely, and actionable. (Level 1a, Grade B)**
2. **Feedback should be based on observed behaviors. (Level 3b, Grade B)**
3. **Both corrective and reinforcing feedback should be provided to learners, although not necessarily at the same time. (Level 4, Grade C)**
4. **Feedback tools are recommended to increase learner satisfaction and volume of feedback; however, the use of tools must be combined with faculty development and a culture of feedback to improve the quality of feedback. (Level 3b, Grade C)**
5. **Feedback should incorporate learner self-assessment. (Level 3b, Grade C)**

### Receiving Feedback

Traditional approaches place learners in the role of passively receiving feedback,^79^,^82^,^176^,^177^ which have been criticized for being too centered on the actions of the instructor. More modern models shift to include the learner as an active participant in soliciting and responding to feedback.^4^,^13^,^153^

### Soliciting Feedback

A crucial initial step to engaging in effective feedback is the act of soliciting feedback that opens the individual to the critiquing process.^55^,^178^ The ability to engage in feedback-seeking behaviors is dependent on four factors: the purpose and quality of the feedback; the learner’s emotional response to feedback; the learner-evaluator relationship; and the workplace culture.^4^,^13^,^50^,^176^,^179^,^180^ While the environment is outside our control, appropriately prepping learners to take contextual factors into account and shifting the focus to environmentally appropriate feedback models may be particularly helpful.^181^,^182^ One common example is the implementation of end-of-shift feedback evaluations. While these have not been identified by faculty as providing a higher quality of feedback, their systematic and reliable delivery results in higher resident satisfaction with the feedback.^32^

### Accepting Feedback

Despite the best intentions of the feedback giver, feedback receptivity is never assured. Literature demonstrates that faculty and learners even disagree on their perceptions of how much feedback is being given.^1^,^16^,^55^ Nevertheless, learner perception significantly impacts feedback acceptance and integration.^130^,^180^,^183^ Different experts have categorized such factors in different ways.^1^,^50^,^57^,^184^ One of the more usable classifications includes categorization of personal (ie, resilience, humility), relational (ie. the strength of supervisory relationship, power differentials), and contextual (ie, culture) factors.^57^

#### Personal Factors

Much of feedback receptivity depends on the learner’s frame of reference. Possessing a growth mindset and employing routine self-reflection is key.^62^,^89^,^96^,^100^,^182^,^183^,^185^–^187^ Learners often approach feedback situations as a performance, probing the situation to see what is expected of them and then acting in a way to better shape their reputation and evaluations.^1^,^13^,^16^,^184^,^188^ Those who have blind spots regarding their weaknesses may be resistant to feedback that challenges their existing self-perception.^130^,^185^–^191^ Failure to internalize feedback happens when a mismatch in external and internally generated assessment occurs. For instance, EM residents consistently assign themselves higher milestone competency ratings than their evaluating attendings.^189^

When feedback is perceived as an attack on personal identity, feedback internalization is effectively hindered. Thus, learners should perceive feedback as opportunities for improvement, rather than statements on character.^1^,^134^,^192^ Evidence suggests that learners educated on feedback have shown comfort in giving and receiving feedback.^105^ Melding self-generated learning goals with faculty-provided observations closes the feedback loop and produces more improved, usable, and well-received feedback aimed at mastering current skills and setting goals for future accomplishments.^12^,^22^,^130^ To bridge the gap between reception of the feedback to internalizing it, multiple experts have outlined various practical tips for learners to use feedback for performance improvement.^190^,^191^,^193^–^200^ We distilled the consistent themes among our recommendations below.

#### Relational Factors

Feedback receptivity is significantly impacted by relational factors such as the strength of the supervisory relationship and power differentials. Regardless of the experience level of the assessor, learners consistently recognize feedback as valid when coming from someone they trust and respect, find credible,^1^,^182^,^192^ and have sought out rather than been assigned,^181^,^197^ such as from role models.^198^,^199^ Mutual respect, establishing shared priorities, and the strength of the educational alliance (defined as the learner’s belief of shared goals, activities, and bonds)^200^ facilitated better feedback receptivity.^57^ Interpersonal skills also affect the relationship and receptivity. Power dynamics and fear of the effect of corrective feedback are barriers to feedback integration.^57^ Learners value feedback when given in a caring, nonjudgmental manner^31^,^62^,^99^ from educators who are friendly and approachable.^201^

#### Contextual Factors

Environmental and cultural considerations affect the receptivity of feedback. The tension between assessment and feedback, specifically the fear of consequences, can lead to learner development of a fixed mindset, limiting growth opportunities.^57^,^96^ For professionalism issues, feedback should be given one on one.^1^,^20^ In busy learning environments, learner-centered approaches grounded in self-directed learning theories (eg, Learner-Centered Approach to Raise Efficiency [L-CARE)] in Clinical Teaching) have been proposed to facilitate more efficient learning.^202^ Ultimately, various studies demonstrated benefit and/or learner preference for standardized,^139^,^203^ structured,^150^,^203^ multisource,^150^ and longitudinal^1^,^105^,^204^,^205^ feedback processes.

## FEEDBACK CULTURE

Feedback culture is defined as written or verbal comments regarding medical knowledge, performance, technique, or patient care within the pedagogical approaches that are routine within a profession.^206^,^207^ The learning culture and type of clinical environment influences learners’ feedback behaviors such as recognizing, seeking, and implementing feedback, namely whether this process is encouraged or not.^99^,^100^ The ED is particularly challenging due to the nature of the work environment, including time constraints, frequent interruptions, and patient acuity, among other factors.^29^,^208^,^209^

**Best Practice Recommendations**

1. **Encourage learners to take an active role in the feedback process. (Level 2b, Grade B)**
2. **Take the work environment into account when creating appropriate feedback systems that are contextually appropriate as a way to improve learner perception of feedback. (Level 2a, Grade B)**
3. **Provide opportunities for learners to build longitudinal trusting relationships in order to promote a strong educational alliance and a growth mindset and to facilitate feedback reception. (Level 4, Grade C)**
4. **Address the tension between assessment and feedback as fear of consequences can predispose a learner to have a fixed mindset, thus limiting learner growth. (Level 4, Grade C)**
5. **Develop and maintain standardized, structured, multisource, and longitudinal feedback processes. (Level 3a, Grade B)**

### Implementation

Institutions should provide and encourage educational opportunities to all individuals involved in feedback interactions including learners and educators. This will allow a culture of growth emphasizing a bidirectional feedback approach^1^,^62^,^100^ with a shift from performance-oriented assessments to learner-oriented feedback.^56^ One method is to emphasize the concept of lifelong learning and normalize the need to identify strengths and weaknesses as a way to grow. Training on giving feedback upward and receiving feedback as an educator can help provide the framework for effective bidirectional feedback.^1^,^16^,^99^,^181^,^194^,^200^ Learners need an environment where vulnerability is acceptable and assessment focuses on a set of shared goals.^14^,^47^ Other strategies include establishing expectations for both educators and learners, promoting specific tasks for all involved, and providing professional development sessions.^57^,^210^ For establishing longitudinal relationships, providing protected faculty time for observational assessments and using standardized feedback tools are beneficial.^97^,^136^,^207^ Furthermore, institutions should encourage a culture of growth. Learners develop a fixed mindset when they perceive performance is linked to assessment, rather than a growth mindset when the relationship is not tied to assessments.^96^

An interdisciplinary, multimodal approach to feedback through MSF can provide additional insight regarding communication, professionalism, and team dynamics and broaden the scope of the feedback received by the learner.^18^,^20^,^121^–^123^,^203^,^211^ Using non-physician medical education specialists to observe learners in the clinical setting may be a useful way to provide tangible feedback on communication, task-switching, professionalism, accountability, and team management skills.^50^

### Barriers to Successful Implementation

Successful implementation of an optimal feedback culture requires a firm understanding of the potential barriers. Grade inflation, discomfort in providing negative feedback, concern with preserving healthy working relationships,^2^,^32^,^84^ time constraints, and personal deficiencies in feedback delivery each present unique challenges.^67^,^84^ Administrative support and the encouragement of the importance of feedback are also important.^67^ While feedback tools may pose a barrier, choosing a user-friendly tool that is of appropriate length and provides sufficient detail with required narrative comments is key.^8^,^9^,^24^

Although limited literature suggests this may be unwarranted,^82^ educators often avoid corrective feedback due to fear of retaliation (especially in systems where learner evaluations are linked to pay or promotion).^80^,^81^ Transparency and focus on the importance of corrective feedback as a learning tool 12 can prevent reluctance to provide negative feedback.^12^,^80^ Finally, a culture of “niceness” can make the learning environment overtly positive, which can hinder the delivery of honest feedback and the creation of a culture of constructive feedback.^12^,^99^,^100^ Being “nice” can be construed as focusing on the positive with a priority on minimizing any negative feelings in the other person, while being “kind” can be construed as focusing on what is best for the learner overall — even if it means creating negative feelings.

### Special Considerations

Implicit bias, which is the unconscious attitudes we have toward people or associated stereotypes, impacts both feedback provided to learners and the perception or receptibility of feedback from faculty.^47^ To minimize this potential bias, assessments should be performed by multiple assessors in multiple different settings.^47^,^63^,^210^ Furthermore, institutions should implement training to identify areas where biases exist, while working to alleviate these biases with full transparency.^47^ Gender bias may lead to different distributions of the frequency and type of feedback. One study found female preceptors completed more feedback forms and provided more corrective feedback to male learners, whereas male preceptors used more communal language and less agentic language with female learners.^212^ Additionally, female learners had more discordant feedback, especially regarding the balance of autonomy and feedback receptivity, than their male counterparts.^213^ Finally, in a study by Stroud, female faculty were found to be perceived as less credible when delivering feedback.^95^

Like racial, cultural, and gender bias, generational gaps can also affect meaningful feedback. Different generations have different patterns of learning. For example, the millennial generation is more engaged in technology and collaborative learning, while preferring clear objectives and timely feedback.^28^,^61^ Additionally, feedback should be provided to all learners, not just low performers. High performers may exhibit the “halo effect,” which can result in them receiving less constructive feedback.^1^ Learner shame responses can be triggered by repeated humiliation experienced in receiving feedback from facilitators. Providing feedback that is focused on behaviors, providing support that normalizes errors in the learning process, and guiding learners through reflection can decrease these learner responses.^60^

**Best Practice Recommendations**

1. **Maximize the impact of feedback by minimizing implicit bias through providing feedback from multiple different assessors in multiple different settings. (Level 4, Grade C)**
2. **Encourage a culture of growth and transparency, focusing on corrective feedback as a learning tool. (Level 4, Grade C)**
3. **Establish expectations for both educators and learners, promote specific tasks for all involved, implement processes to encourage bi-directional feedback, and provide development sessions for professional growth. (Level 4, Grade C)**
4. **Shift emphasis from performance-oriented assessment to learner-oriented feedback. (Level 2b, Grade B)**

## LIMITATIONS

Although we performed a comprehensive search guided by a medical librarian in conjunction with a bibliographic review and expert consultation to augment content when needed, we used a single search engine (MEDLINE), and it is possible that we may have missed some pertinent articles. In instances where evidence in the form of high-quality data was limited or lacking, we relied upon expert opinion and group consensus for the best practice recommendations. The literature specific to feedback for the field of EM and within graduate medical education is limited. To supplement, we included relevant articles from other medical specialties and health-related professions as we believe that EM, as a specialty, can learn from other colleagues across many disciplines. Finally, in areas where evidence was not available, we used the consensus from the expertise of our authorship group. While our author group possesses experience in research and scholarship in both feedback and medical education, there is a potential for bias to be introduced during this process. Therefore, we also sought peer review from the CORD Best Practices Subcommittee and posted it online for open review feedback by the CORD community.

## CONCLUSION

Feedback is integral to professional development. This paper provides readers with guidance on the use of feedback in medical education, with a focus on instructor strategies for giving feedback, learner strategies for receiving feedback, and suggestions for fostering a culture of feedback.

## Supplementary Information



## Figures and Tables

**Table 1:** Oxford Centre for Evidence-Based Medicine Levels of Evidence.^42^

Level of evidence	Definition
1a	Systematic review of homogenous RCTs
1b	Individual RCT
2a	Systematic review of homogenous cohort studies
2b	Individual cohort study or a low-quality RCT*
3a	Systematic review of homogenous case-control studies
3b	Individual case-control study**
4	Case series/Qualitative studies or low-quality cohort or case-control study***
5	Expert/consensus opinion

* Defined as <80% follow up;

** includes survey studies and cross-sectional studies;

*** defined as studies without clearly defined study groups.

*RCT*, randomized controlled trial.

**Table 2:** Oxford Centre for Evidence-Based Medicine Grades of Recommendation.^42^

Grade of evidence	Definition
A	Consistent level 1 studies
B	Consistent level 2 or 3 studies or extrapolations* from level 1 studies
C	Level 4 studies or extrapolations* from level 2 or 3 studies
D	Level 5 evidence or troublingly inconsistent or inconclusive studies of any level

* Extrapolation refers to data used in a situation that has potentially clinically important differences than the original study situation.

**Table 3:** Feedback techniques.

Feedback techniques			

	Description	Types	Pearls & pitfalls
Direct observation	Real-time, one-on-one observation and feedback of a learner for both clinical and non-clinical skills, either in the clinical setting, simulation, or nonclinical environment	Objective Structured Clinical Examination (OSCE)^95^	Formative and timely but time- and resource-intensive
		Observed Structured Teaching Exercises (OSTE)^115^,^119^,^144^	Learner-centered
		Structured clinical observation shadowing	Beware of the “Hawthorne effect” Time intensive

Real-time feedback	Getting feedback to the learner at the moment, whether verbal, written or using an app or virtual form	Online survey (eg, Google Forms, Qualtrics, SurveyMonkey)	Learner-centered, Improves quantity of feedback
		EMR based^68^	May be challenging to give corrective feedback
		One minute mentor^145^	
		Minute feedback system^27^,^138^	

Self-assessment	Learners reflect on, diagnose, and critique their own progress; often informs learning goals to mark intended outcomes	Johari window^99^,^100^	Feedback can be focused on intended goals
		Reflective feedback conversation^74^	Caution on only focusing on self-assessed topics, as self-assessment may not identify all learner needs

Evaluative models	Framework for assessing learners based on established categories such as competencies or entrustable professional activities	CanMEDS^140^	Focused feedback
		Evaluation and feedback for effective clinical teaching instrument (EFFECT) tool^146^	Snapshot in time
		Entrustable professional activities (EPA)^141^,^147^	Blurs line between assessment and feedback
		ACGME milestones^18^,^148^	Limits narrative feedback
		Inviting co-workers to evaluate Physicians tool (INCEPT)^124^	Formative feedback Through a survey with similar questions to different respondents (ie, groups of peers, coworkers, and residents)
		Mini peer assessment tool (Mini-PAT)	
		Team assessment of behavior (TAB)^112^	Needs many encounters to be reliable
		Emergency medicine humanism scale (EM-HS)^121^,^122^	TAB is primarily a free-text tool
			EM-HS MSF tool from nursing and faculty
		Communication assessment tool (CAT)^129^	Often surface-level feedback only

Specialized feedback techniques	Various techniques for in-the-moment feedback, sometimes combining acquiring clinical information along with giving feedback	Relationship, Reaction, Content, Change (R2C2) model^86^,^152^,^153^	Quick/efficient for a variety of learners
		Ask-Tell-Ask^154^	Built-in mechanism for feedback
		One minute preceptor^39^,^143^	
		Summarize the history and physical, narrow differential, analyze options, probe, plan management, self-directed learning (SNAPPS)^39^,^143^	Promotes learner accountability
			Feedback sandwich falls short of a reflective conversation as recipients learn to ignore positive statements because they know a “but” is coming.
		Setting, Probe, Inquire, Knowledge, Empathy, Summary (SPIKES)^104^	Concise framework that allows gentle probing of the learner to commit, while then allowing timely, specific, actionable feedback to be given.
		Professionalism & Procedural Skills, Reporter, Interpreter, Manager, Educator, Procedural skills (PRIMES)^22^,^23^	Process is facilitated with an iPad app called PRIMES with residents’ self-assessment and goal setting. The faculty then assesses the resident blindly. The app compares assessment with results visually highlighting areas of agreement and disagreement.
		Creating an environment, observing/preparing for feedback, assembling the learner and providing feedback, check/follow-up afterwards (COACH)^91^	Can be applicable across a variety of medical disciplines and learning environments, simultaneously teaches both the giving and eliciting of feedback
		Pendleton’s Model of Feedback^74^	Techniques must be learned

*ACGME*, Accreditation Council for Graduate Medical Education; *CANMeds*, Royal College of Physicians and Surgeons of Canada competency framework; *EMR*, electronic medical record.

**Table 4:** Feedback tools.

	Name	Description	Examples
Physical			
	Feedback Cards^32^,^155^,^156^,^158^,^159^,^173^	This tool is typically handed out by the learner and often designed to identify areas the learner desires feedback on.	Encounter cards, debrief cards, “Prescription pads” feedback cards, pocket feedback
	Direct Observation Cards^88^,^102^,^160^–^162^	This tool uses direct observation and performance assessment with written narrative feedback.	Mini Direct Observation (Mini-Card)
			Mini Clinical Evaluation Exercise (Mini-CEX)
	Field note tool^174^	This written tool with open-ended questions for both the learner and the assessor to facilitate a two-way discussion and real-time workplace-based assessment with the development of action plans.	Field note tool
	Multisource feedback tools^112^,^121^,^122^,^124^,^129^,^142^	Techniques aimed at gathering feedback from various assessors to give a more comprehensive view of the learner.	INCEPT, Mini-PAT, TAB, EM-HS, CAT
E-tools			
	Web-based^27^,^138^,^145^,^163^–^165^,^175^	Designed to take a minute to complete in order to facilitate same-day, timely responses in brief narrative comments, these systems were felt to be easy to institute and feasible approach to assessing students, particularly regarding professionalism behavior. These online survey platforms can increase the amount and timeliness of feedback. However, there is a need to emphasize data consolidation and distribution with these tools to ensure that feedback is distributed in a timely manner.	Facebook Dashboard, QuickNotes, TIPreport, One Minute Mentor/Minute Feedback System, and online surveys such as Google Forms and SurveyMonkey
	App-based^18^,^70^,^168^–^170^	This is a feedback tool accessed through a mobile application to allow ease of use. These apps were shown to help collect useful data and provide an increased amount of quality feedback. They also were found to have benefits of accessibility, low cost, and ability to trend resident progression.	Mobile Medical Milestones Application (M3App), Healthcare Supervision Logbook App, System for improving and measuring procedural learning (SIMPL), Resident report card (RRC), MyTIPReport
	Online Social Media Platforms^18^,^171^	Use of social media platforms to allow discussion and feedback through the internet to obtain feedback through crowdsourcing. Online social media platforms can focus on in-the-moment discussion points and provide easily digestible feedback from a diverse group of evaluators.	Twitter, Instagram, Facebook
	Video Recording^43^,^103^,^133^,^172^	This form can play a role as a feedback tool in itself and as an adjunct with other feedback tools such as checklists. By recording learners and educators in various situations evaluators can provide specific guidance afterward.	Pre-recorded clinical, feedback sessions, educational, simulation sessions, OSTEs, OSCEs, etc

*INCEPT*, Inviting Co-workers to Evaluate Physicians Tool; *Mini-PAT*, Mini Peer Assessment Tool; *TAB*, Team Asessment of Behavior; *EM-HS*, Emergency Medicine Humanism Scale; *CAT*, Communication Assessment Tool; *OSTE*, Observed Structured Teaching Exercises; *OSCE*, Objective Structured Clinical Examination.

## CORD Best Practice Committee 2020–2021

**Michael Gottlieb, MD – Co-Chair**
  *Rush University Medical Center*
**Sreeja Natesan, MD – Co-Chair**
  *Duke University*
**Brian Barbas, MD**
  *Loyola University*
**Jennie Buchanan, MD**
  *Denver Health Medical Center*
**Richard Byyny, MD**
  *Denver Health Medical Center*
**Guy Carmelli, MD**
  *University of Massachusetts Medical School*
**Molly Estes, MD**
  *Loma Linda University*
**Katja Goldflam, MD**
  *Yale University*
**Andrew Grock, MD**
  *University of California - Los Angeles*
**Jaime Jordan, MD**
  *University of California - Los Angeles*
**Deena Khamees, MD**
  *University of Michigan*
**Andrew King, MD**
  *The Ohio State University*
**Krystin Miller, MD**
  *The Ohio State University*
**Melissa Parsons, MD**
  *University of Florida - Jacksonville*
**Alexander Sheng, MD**
  *Boston Medical Center*
**Brian Wood, MD**
  *St. Joseph’s Medical Center*
**Saadia Akhtar, MD**
  *Mount Sinai*
