# Supplementary material for: Feedback in Medical Education: An Evidence-based Guide to Best Practices from the Council of Residency Directors in Emergency Medicine
Source: West J Emerg Med. 2023 May 5;24(3):479–94. doi: 10.5811/westjem.56544 (PMC10284500; doi:10.5811/westjem.56544)
Supplement: Supplementary file 1 [file wjem-24-479-s001.docx]

**(((((((((((((feedback) AND (registrar OR registrars OR graduate medical education OR interns OR intern OR resident OR residents OR house staff OR residency))) AND (strategies OR techniques OR methods OR guidelines[tiab] OR "best practices"[tiab]))) AND (teaching OR teachers OR educators OR educator OR supervisor OR supervisors OR supervising OR directors[tiab] OR director[tiab]))) AND ((providing OR giving OR receiving OR provision OR provided OR provide OR provides OR given OR gave OR give OR effective OR effectively OR receiving OR receive OR received OR receives)))))) AND (emergency medicine OR hospital medicine OR clinical medicine OR clinic OR clinical OR clinics OR hospital OR hospitals OR emergency OR emergencies)))**
